# Effect of sucralfate suspension gel on gastroscopic pathology and inflammatory cytokines in patients with Helicobacter pylori-positive chronic non-atrophic gastritis

**DOI:** 10.12669/pjms.42.2.12890

**Published:** 2026-02

**Authors:** Gang Liu, Pinna Yang, Shaohua Wang, Quanxi Liu

**Affiliations:** 1 Gang Liu Department of Gastroenterology, Beijing Hospital of Integrated Traditional Chinese and Western Medicine, Beijing 100039, Beijing, China; 2 Pinna Yang Clinical Laboratory, Beijing Hospital of Traditional Chinese Medicine, Capital Medical University, Beijing 100039, Beijing, China; 3 Shaohua Wang Department of Gastroenterology, Beijing Hospital of Integrated Traditional Chinese and Western Medicine, Beijing 100039, Beijing, China; 4 Quanxi Liu Department of Gastroenterology, Beijing Hospital of Integrated Traditional Chinese and Western Medicine, Beijing 100039, Beijing, China

**Keywords:** Amoxicillin, Bismuth potassium citrate, Chronic non-atrophic gastritis, Gastroscopic pathology, Helicobacter pylori infection, Inflammatory cytokine, Sucralfate suspension gel

## Abstract

**Objectives:**

To investigate the effects of sucralfate suspension gel on gastroscopic pathology and inflammatory cytokine levels in patients with *Helicobacter pylori* (Hp)-positive chronic non-atrophic gastritis.

**Methodology:**

A retrospective analysis was conducted on 80 outpatients treated at Beijing Hospital of Integrated Traditional Chinese and Western Medicine between January 2022 to January 2025. Patients were divided into a control group (*n =* 40) and an observation group (*n =* 40). The control group received standard quadruple therapy, while the observation group received additional treatment with sucralfate suspension gel on top of the standard regimen. Clinical outcomes, gastroscopic pathological scores, gastrointestinal hormone levels, inflammatory cytokine levels and incidence of adverse events were compared between the two groups.

**Results:**

The observation group demonstrated a significantly higher overall response rate compared with the control group(*P<* 0.05). Post-treatment gastroscopic pathological scores were significantly lower in the observation group than in the control group(*P<* 0.05). Levels of gastrointestinal hormones were significantly elevated in the observation group(*P<* 0.05). Furthermore, post-treatment levels of interleukin-2, matrix metalloproteinase-9 and tumor necrosis factor-α were significantly reduced in the observation group compared with the control group(*P<* 0.05, respectively). The incidence of adverse events did not differ significantly between the two groups(*P>* 0.05).

**Conclusion:**

The addition of sucralfate suspension gel to standard therapy in the treatment of Hp-positive chronic non-atrophic gastritis yields favorable clinical outcomes. It can significantly improve gastroscopic pathology, reduce inflammatory cytokine levels and enhance gastrointestinal hormone secretion without compromising treatment safety.

## INTRODUCTION

Chronic non-atrophic gastritis(CNG) is a common gastrointestinal disorder characterized primarily by epigastric pain, bloating and acid regurgitation. These manifestations are typically attributed to prolonged irritation of the gastric mucosa, i.e., the protective lining of the stomach, triggered by irregular eating habits, psychological stress or bacterial infection. This sustained mucosal irritation results in the infiltration of immune cells into the superficial layers of the gastric lining, leading to inflammation. Clinically, this condition has also been referred to as superficial gastritis.^1^ *Helicobacter pylori* (Hp), a Gram-negative bacterium that colonizes the gastric mucosa, is considered the most prevalent etiological agent of CNG. Through extensive replication in the gastric environment, Hp induces chronic inflammation and damages the mucosal tissue.^2^

Patients with CNG commonly present with upper gastrointestinal symptoms such as epigastric distension, pain, belching and acid reflux. These may be accompanied by systemic signs, including loss of appetite, weight loss and anemia. In severe or prolonged cases, the condition may progress to atrophic gastritis, which is associated with an elevated risk of gastric carcinogenesis.^3^ The mainstay of treatment for CNG involves pharmacological strategies aimed at eradicating Hp, suppressing gastric acid secretion and protecting the gastric mucosa. Among these, quadruple therapy comprising antibiotics and proton pump inhibitors is most frequently used and has proven effective in eradicating Hp while minimizing adverse effects.^4^

However, increasing clinical evidence suggests that standard quadruple therapy alone may not achieve optimal therapeutic outcomes in all patients with Hp-positive CNG. Sucralfate, a mucosal protective agent with additional antacid properties, forms a physical barrier over injured gastric mucosa and has demonstrated favorable therapeutic effects in the treatment of duodenal ulcers and stress-related gastric lesions.^5^ Against this backdrop, this study investigated the adjunctive use of sucralfate suspension gel combined with standard quadruple therapy in patients with Hp-positive CNG, aiming to evaluate its effects on gastroscopic pathology and inflammatory cytokine profiles.

## METHODOLOGY

A retrospective analysis was conducted on 80 outpatients diagnosed with Hp-positive CNG and treated at Beijing Hospital of Integrated Traditional Chinese and Western Medicine between January 2022 to January 2025. Patients were equally divided into two groups: a control group and an observation group (*n =* 40 each). In the observation group, there were 24 males and 16 females, aged 19-63 years (mean age: 49.61 ± 7.61 years). Body mass index (BMI) ranged from 18 to 27 kg/m^2^ (mean: 22.24 ± 2.81 kg/m^2^) and disease duration ranged from 3-12 months (mean: 6.98 ± 1.89 months). In the control group, there were 21 males and 19 females, aged 18-65 years (mean age: 50.94 ± 7.25 years). BMI ranged from 18 to 28 kg/m^2^ (mean: 22.64 ± 2.98 kg/m^2^) and disease duration ranged from 4-11 months (mean: 7.24 ± 1.91 months). There were no statistically significant differences in baseline characteristics between the two groups (all *P >* 0.05).

### Ethical approval:

The study was approved by the Institutional Ethics Committee of Beijing Hospital of Integrated Traditional Chinese and Western Medicine (No: ZXYEC-KT-2022-03-P04; date: November 19, 2022) and written informed consent was obtained from all participants.

### Inclusion criteria:

- Confirmed Hp infection based on the¹^3^C-urea breath test in accordance with the *Guideline for Primary Care of Helicobacter pylori Infection*.^6^
- Diagnosis of CNG confirmed by gastroscopic histopathology, consistent with the *Guideline for Primary Care of Chronic Gastritis*.^7^
- No known allergy to any components of the quadruple therapy regimen or sucralfate.
- Provided informed consent for both the treatment protocol and study.

### Exclusion criteria:

- Coexisting gastric diseases such as peptic ulcer, gastric perforation, gastrointestinal bleeding or gastric cancer.
- Use of any additional medications during the study period.
- Non-adherence to the prescribed treatment regimen.
- Discontinuation of treatment without medical advice
- Incomplete clinical or laboratory data required for outcome assessment.

### Treatment methods:

The control group received standard quadruple therapy as follows:

- Rabeprazole enteric-coated capsules (Specification: 20 mg; CFDA Approval No. H20234448; YaoPharma Co., Ltd., Chongqing, China), taken orally, 20 mg twice daily;
- Amoxicillin capsules (Specification: 0.25 g; CFDA Approval No. H33021381; Zhejiang Jinhua CONBA Bio-pharm. Co., Ltd., Zhejiang, China), taken orally, 1.0 g twice daily;

Clarithromycin tablets (Specification: 0.25 g; CFDA Approval No. H20083281; Zhejiang Better Pharmaceuticals Co., Ltd., Zhejiang, China), taken orally, 0.5 g twice daily; (4) Bismuth potassium citrate capsules (Specification: 0.3 g; CFDA Approval No. H10920098; Livzon Pharmaceutical Group Inc., Zhuhai, China), taken orally, 0.6 g twice daily. The treatment duration was 14 consecutive days. The observation group received the same quadruple therapy regimen for 14 days, followed by adjunctive treatment with sucralfate suspension gel (Specification: 5 ml: 1 g; CFDA Approval No. H20080322; Kunming Jida Pharmaceutical Co., Ltd., Yunnan, China), administered orally at a dose of 5ml two times daily before breakfast and before bedtime on an empty stomach, for an additional 18 consecutive days.

### Outcome measures:

Comparison of Clinical Efficacy: According to the *Chinese Consensus on Family-based Helicobacter pylori Infection Control and Management* (2021)^8^, clinical efficacy was evaluated using the following criteria:

### Significant Response (SR):

Complete resolution of clinical symptoms, negative Hp status upon retesting and a reduction in gastroscopic pathological score >80%;

### Partial Response (PR):

Moderate improvement in symptoms, negative Hp status upon retesting and a reduction in gastroscopic pathological score by 30%-80%.

#### No Response:

No improvement in symptoms, persistent Hp positivity and gastroscopic pathological score reduction <30%. The overall response rate (ORR) was calculated as: ORR (%) = (SR + PR) / Total number of cases × 100%.

### Comparison of Gastroscopic Pathological Score:

Before and after treatment, all patients underwent gastroscopy using the Fujifilm BL-7000 electronic endoscope (Fujifilm Corporation, G.X.Z.J. No. 20182060487). Biopsy specimens from the upper GI track were collected from different pathologies seen during endoscopy and were stored at -80. Gastroscopic findings were assessed in correlation with histopathological results, with focus on the following features of the gastric mucosa: erythema, erosion, inflammatory activity and intramucosal hemorrhage. Each of the four pathological indicators was scored on a four-point scale: 0 = none, 1 = mild, 2 = moderate, 3 = severe.^9^

Comparison of Gastrointestinal Hormone Levels: Fasting venous blood samples (3 mL) were collected from all patients before and after treatment. Samples were centrifuged using a VH-10FB high-speed medical centrifuge (Hunan Benock Instrument & Equipment Co., Ltd.; X.C.X.B. No. 20221483) to isolate and harvest serum. The serum levels of gastrointestinal hormones, including gastrin (GAS), vasoactive intestinal peptide (VIP) and motilin (MOT), were measured using enzyme-linked immunosorbent assay. The assay was performed on a DSX100 multifunctional microplate reader (G.S.Y.J.X. (J) No. 2013-2402416; Collagen Matrix, Inc., Beijing, China), with test kits supplied by Xiamen Biosino-Agiaccu Biotechnology Co., Ltd.

### Comparison of Inflammatory Cytokine Levels:

The same serum samples were used to assess the levels of matrix metalloproteinase-9 (MMP-9), interleukin-2 (IL-2) and tumor necrosis factor-alpha (TNF-α) using a fluorescence immunoassay. Measurements were performed using the AutoTRFIA-8 fully automated fluorescence immunoassay analyzer (Y.X.Z.Z. No. 20172401890; Guangzhou Fenghua Bioengineering Co., Ltd., Guangdong, China), with assay kits purchased from Shanghai Huashi Asia-Pacific Bio-Pharmaceutical Co., Ltd. Comparison of Treatment Safety: The incidence of adverse reactions during the treatment period was recorded and compared between the two groups to evaluate treatment safety.

### Statistical analysis:

All statistical analyses were conducted using SPSS 25.0. Measurement data were expressed as mean ± standard deviation (*x̄±s*) and count data were presented as frequencies and percentages (*n*[%]). Comparisons between groups were made using the *t*-test for continuous variables and the chi-square (χ^2^) test for categorical variables. To address the issue of multiple comparisons, the Bonferroni method was used to adjust the significance level (α = 0.05 / number of comparisons). All reported *p-values* are the results after correction. A *p-values* <0.05 was considered statistically significant.

## RESULTS

The ORR in the observation group was significantly higher than that in the control group (*P <* 0.05) Table-I. After treatment, the gastroscopic pathological scores for mucosal erythema, mucosal erosion, inflammatory activity and intramucosal hemorrhage were all significantly lower in the observation group than in the control group (all *P <* 0.05) Table-II. The post-treatment levels of GAS, VIP and MOT were significantly higher in the observation group compared with the control group (*P <* 0.05, respectively) Table-III.

**Table-I:** Comparison of clinical efficacy [n(%)].

Group	n	SR	PR	NR	ORR
Observation	40	23(57.50)	14(35.00)	3(7.50)	37(92.50)
Control	40	18(45.00)	12(30.00)	10(25.00)	30(75.00)
*χ^2^ value*					4.501
*P-value*					0.034

**Table-II:** Comparison of gastroscopic pathological scores (*χ̅*±*S*).

Group	n	Mucosal erythema		Mucosal erosion		Inflammatory activity		Intramucosal hemorrhage	
		Pre-treatment	Post-treatment	Pre-treatment	Post-treatment	Pre-treatment	Post-treatment	Pre-treatment	Post-treatment
Observation	40	2.53±0.41	0.64±0.15*	2.32±0.43	0.72±0.16*	2.49±0.48	0.79±0.20*	2.29±0.51	0.86±0.21*
Control	40	2.50±0.45	0.85±0.19*	2.38±0.48	0.94±0.22*	2.41±0.50	1.03±0.25*	2.35±0.52	1.12±0.27*
*t-value*		0.320	5.607	0.604	5.215	0.750	4.847	0.536	4.910
*P-value*		0.750	0.000	0.547	0.000	0.455	0.000	0.594	0.000

***Note:*** Compared with pre-treatment values in the same group,

* P < 0.05 (This also applies to other tables in the remainder of the manuscript.)

**Table-III:** Comparison of gastrointestinal hormone levels (*χ̅*±*S*).

Group	n	GAS(ng/ml)		VIP(ng/ml)		MOT(mg/ml)	
		Pre-treatment	Post-treatment	Pre-treatment	Post-treatment	Pre-treatment	Post-treatment
Observation	40	111.42±13.14	215.22±22.32*	97.11±10.62	189.29±18.34*	251.56±28.14	386.28±37.42*
Control	40	115.89±16.19	198.01±20.98*	95.81±11.02	167.11±17.44*	249.01±27.23	350.63±34.61*
*t-value*		1.387	3.663	0.552	5.712	0.424	4.562
*P-value*		0.169	0.000	0.582	0.000	0.672	0.000

The post-treatment levels of the inflammatory cytokines IL-2, MMP-9 and TNF-α were significantly lower in the observation group compared with the control group (*P <* 0.05, respectively) Table-IV. There was no statistically significant difference in the incidence of adverse events between the two groups (*P >* 0.05) Table-V.

**Table-IV:** Comparison of inflammatory cytokine levels (*χ̅*±*S*).

Group	n	IL-2(ng/ml)		MMP-9(ng/ml)		TNF-α(mg/ml)	
		Pre-treatment	Post-treatment	Pre-treatment	Post-treatment	Pre-treatment	Post-treatment
Observation	40	82.34±9.36	44.21±5.62*	37.11±5.12	19.09±3.54*	121.56±13.14	66.28±7.42*
Control	40	83.78±9.27	49.97±5.89*	38.36±5.26	23.41±4.14*	122.63±12.59	74.02±8.01*
*t-value*		0.712	4.598	1.107	5.137	0.505	4.483
*P-value*		0.479	0.000	0.271	0.000	0.615	0.000

**Table-V:** Comparison of adverse event rates (n[%]).

Group	n	Nausea and vomiting	Diarrhea and abdominal pain	Constipation	Abnormal liver function	Overall Incidence
Observation	40	1(2.50)	1(2.50)	1(2.50)	1(2.50)	4(10.00)
Control	40	1(2.50)	1(2.50)	1(2.50)	0(0.00)	3(7.50)
*χ^2^ value*						0.157
*P-value*						0.692

## DISCUSSION

In this study, the observation group received sucralfate suspension gel in addition to conventional quadruple therapy, resulting in significantly improved clinical outcomes compared with the control group. These findings suggest that sucralfate is a valuable adjunctive agent in the treatment of Hp-positive CNG. Sucralfate suspension gel is a potent gastric mucosal protectant. Its therapeutic benefits are primarily attributed to its unique mechanism of action. Upon oral administration, sucralfate dissociates in the acidic gastric environment to release negatively charged sucrose sulfate complexes. These anionic compounds bind strongly to positively charged proteins on the surface of the damaged mucosa (*e.g*., exuded plasma proteins, necrotic tissue), forming an insoluble, viscoelastic gel layer. This barrier physically separates the injured mucosa from aggressive factors such as gastric acid, pepsin and bile, thereby exerting a protective effect. Notably, the adhesive strength of sucralfate to damaged mucosa is approximately six times greater than its affinity for healthy mucosa, offering sustained protection and promoting mucosal healing.^10^ Moreover, sucralfate has been shown to stimulate the endogenous mucosal defense and repair mechanisms, including the mucus-bicarbonate barrier. It enhances prostaglandin E_2_ synthesis, which increases mucus viscosity and hydrophobicity, while also promoting bicarbonate secretion, thereby augmenting the mucosa’s resistance to acid injury.^11^ Additionally, sucralfate upregulates the expression and activity of key repair mediators such as epidermal growth factor and fibroblast growth factor, facilitating their accumulation in ulcerated or atrophic regions, where they promote epithelial regeneration. These multifaceted protective and regenerative properties explain the enhanced therapeutic efficacy observed when sucralfate is combined with quadruple therapy.^12^ Post-treatment gastroscopic pathological scores for mucosal erythema, mucosal erosion, inflammatory activity and intramucosal hemorrhage were significantly improved in the observation group. This highlights sucralfate’s role in creating a favorable environment for mucosal healing, ultimately contributing to improved histopathological outcomes in patients with Hp-positive CNG.

Patients with Hp-positive CNG often experience gastrointestinal dysregulation due to symptoms such as belching and acid reflux. These disturbances are frequently accompanied by abnormal fluctuations in gastrointestinal hormone levels. Among these, GAS, VIP and MOT are well-established indicators of gastrointestinal endocrine function. GAS, secreted by G cells, stimulates gastric antral motility and intestinal activity.^13^ VIP, a polypeptide released by enteric neurons, relaxes intestinal smooth muscle and facilitates gastric emptying by reducing intragastric retention.^14^ MOT, a key gastrointestinal peptide hormone, promotes intestinal peristalsis and accelerates the transit of intestinal contents.^15^

In this study, the post-treatment levels of GAS, VIP and MOT were significantly higher in the observation group compared with the control group, indicating that the adjunctive use of sucralfate suspension gel may contribute to the normalization of gastrointestinal hormone levels. This might be explained by sucralfate’s robust mucosal protective properties and its paracrine regulatory activity. Sucralfate may influence the function of G cells (which secrete GAS) and Mo cells (which secrete MOT), thereby improving gastrointestinal hormone levels.^16^ Another important aspect of the pathogenesis of CNG is the inflammatory response, which is characterized by elevated levels of pro-inflammatory cytokines. IL-2, MMP-9 and TNF-α are commonly elevated in patients with this condition and are key mediators of mucosal inflammation.^17^

CNG is a common condition in gastroenterology, with a relatively high prevalence in clinical practice. Epidemiological studies indicate that its incidence in the adult population in China ranges from 5% to 10%, with a higher occurrence among middle-aged and elderly individuals. Current research on its pathogenesis has identified Hp infection as the most common etiological factor. It is estimated that Hp-positive cases account for 60%-70% of patients with CNG.^18^ The underlying mechanism primarily involves extensive colonization of the gastric mucosa by Hp, which compromises the integrity of the mucosal barrier. This disruption leads to an imbalance in the gastric mucosal defense system, resulting in excessive gastric acid secretion that exacerbates mucosal injury. When the extent of this injury exceeds the mucosa’s inherent capacity for self-repair, it gives rise to persistent epithelial damage and ultimately the development of CNG. In addition to Hp infection, several other risk factors contribute to disease onset and progression, including poor dietary habits, unhealthy lifestyle behaviors, smoking, alcohol consumption and immune dysfunction. Conventional treatment strategies for CNG primarily focus on the eradication of Hp, suppression of gastric acid secretion and enhancement of mucosal protection.^19^ Among these, the quadruple therapy regimen comprising antibiotics and proton pump inhibitors remains the first-line treatment for Hp-positive cases. In this study, the control group, which received standard quadruple therapy, exhibited an ORR of 75.00%, indicating that while effective, this regimen still leaves room for therapeutic improvement.

In the present study, the observation group demonstrated significantly lower levels of these inflammatory markers compared with the control group, suggesting that sucralfate may effectively suppress inflammatory responses. This effect is thought to be mediated by sucralfate’s ability to adsorb bile acids and pepsin-thereby reducing their irritant effect on the mucosa-as well as by its stimulation of endogenous protective factors, inhibition of neutrophil activation and suppression of inflammatory cytokine release.^20^ In terms of safety, the incidence of adverse events did not significantly differ between the two groups, indicating that the addition of sucralfate to the quadruple therapy regimen did not compromise treatment safety.

### Limitations:

A small number of samples is the limitation of the study. As a retrospective study, this study cannot completely exclude the effect of sucralfate suspension gel on gastroscopic pathology and inflammatory cytokine levels in patients with Hp-positive chronic non-atrophic gastritis, prospective studies are needed to establish temporality between them. Despite the above limitations, this study provides important empirical data on the combination of sucralfate suspension gel with standard quadruple therapy demonstrates substantial clinical value in the treatment of Hp-positive CNG.

## CONCLUSIONS

The combination of sucralfate suspension gel with standard quadruple therapy demonstrates substantial clinical value in the treatment of Hp-positive CNG. This therapeutic approach can effectively improve gastroscopic pathological scores, reduce inflammatory cytokine levels, restore gastrointestinal hormone balance and enhance overall treatment safety.

### Authors’ Contributions:

**GL:** Carried out the studies, data collection, drafted the manuscript and are responsible and accountable for the accuracy or integrity of the work.

**PY:** Performed the statistical analysis and participated in its design. Critical review.

**SW** and **QL:** Literature search, participated in acquisition, analysis or interpretation of data and drafted the manuscript.

All authors read and approved the final manuscript.
